# The past, present and future of mitochondrial genomics: have we sequenced enough mtDNAs?

**DOI:** 10.1093/bfgp/elv027

**Published:** 2015-06-27

**Authors:** David Roy Smith

**Keywords:** genome sequencing, microbial diversity, mitochondrial genome, mitochondrial transcriptome, Marine Microbial Eukaryotic Transcriptome Sequencing Project

## Abstract

The year 2014 saw more than a thousand new mitochondrial genome sequences deposited in GenBank—an almost 15% increase from the previous year. Hundreds of peer-reviewed articles accompanied these genomes, making mitochondrial DNAs (mtDNAs) the most sequenced and reported type of eukaryotic chromosome. These mtDNA data have advanced a wide range of scientific fields, from forensics to anthropology to medicine to molecular evolution. But for many biological lineages, mtDNAs are so well sampled that newly published genomes are arguably no longer contributing significantly to the progression of science, and in some cases they are tying up valuable resources, particularly journal editors and referees. Is it time to acknowledge that as a research community we have published enough mitochondrial genome papers? Here, I address this question, exploring the history, milestones and impacts of mitochondrial genomics, the benefits and drawbacks of continuing to publish mtDNAs at a high rate and what the future may hold for such an important and popular genetic marker. I highlight groups for which mtDNAs are still poorly sampled, thus meriting further investigation, and recommend that more energy be spent characterizing aspects of mitochondrial genomes apart from the DNA sequence, such as their chromosomal and transcriptional architectures. Ultimately, one should be mindful before writing a mitochondrial genome paper. Consider perhaps sending the sequence directly to GenBank instead, and be sure to annotate it correctly before submission.

## Introduction

I just finished peer-reviewing another mitochondrial genome paper—the fourth in as many weeks. This time it was a manuscript describing a half-dozen new mitochondrial DNA (mtDNA) sequences from a poorly studied algal lineage. These days, the mtDNA review requests are arriving faster than I can turn them out, which is disturbing. Next-generation sequencing (NGS) techniques and sophisticated bioinformatics programs have made it quick, easy and cheap to sequence and assemble entire mitochondrial genomes from almost any eukaryotic species for which total DNA can be isolated. In most instances, you do not even need to purify mitochondria or do a tedious mtDNA isolation through cesium chloride density gradient centrifugation [1] before sequencing. A single run of whole genomic DNA on an NGS platform, such as Illumina’s HiSeq 2000 sequencing system, typically yields enough mtDNA-derived reads to assemble, with high coverage, the complete mitochondrial genome or, in the case of complementary DNA, the entire mitochondrial transcriptome [2, 3].

The high copy number and elevated expression levels of mitochondrial genomes mean that they represent a significant proportion (up to 25%) of the reads generated from next-generation DNA and RNA sequencing (DNA- and RNA-Seq) experiments, respectively [2–4]. And because mitochondrial transcripts are typically rich in adenine and thymine, and in some lineages polyadenylated [5], their contribution to the overall number of reads has been shown to go up with increased poly-A RNA selection [4]. Moreover, the small size, reduced gene content and compact nature of some mitochondrial chromosomes can result in straightforward genome assemblies and annotation (with notable exceptions [6–8]). There are even a number of free, online bioinformatics resources devoted to analysing mtDNAs, such as MFannot—an automated annotation tool for mitochondrial genomes, which requires little if any manual corrections [9]. Consequently, mtDNAs are presently the most sequenced type of eukaryotic chromosome [10].

**Figure 1:**
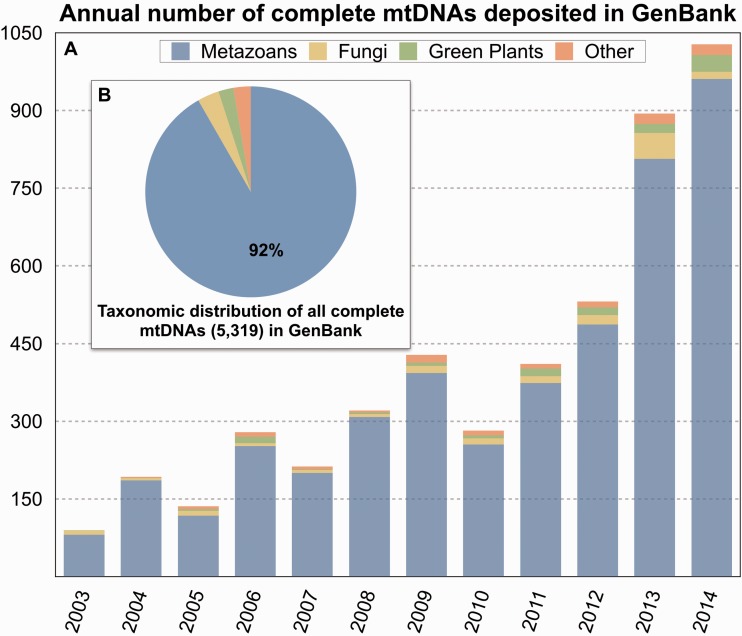
Complete mitochondrial genome sequences in GenBank. (**A**) Annual number deposited since 2003. (**B**) Total number of sequences (5319) as of 5 February 2015. Statistics from the National Center for Biotechnology Information Organelle Genome Resources [53]. (A colour version of this figure is available online at: http://bfg.oxfordjournals.org)

As of 1 February 2015, there were more than 5300 complete mtDNAs in the National Center for Biotechnology Information databank, also called GenBank, which is greater than the number of unique bacterial, viral or nuclear genomes. And when considering different sequences for the same species, there are over 35 000 mtDNA entries. Moreover, the rate of mitochondrial genome sequencing is growing exponentially (Figure 1). In 2014, more than a thousand mtDNAs were deposited in GenBank, which is twice the number from 2012 and almost four times that from 2010 (Figure 1). And in the first 2 months of 2015, nearly 200 animal mtDNAs were sequenced, indicating that the current year will be even more fruitful for mitochondrial genomics.

**Figure 2:**
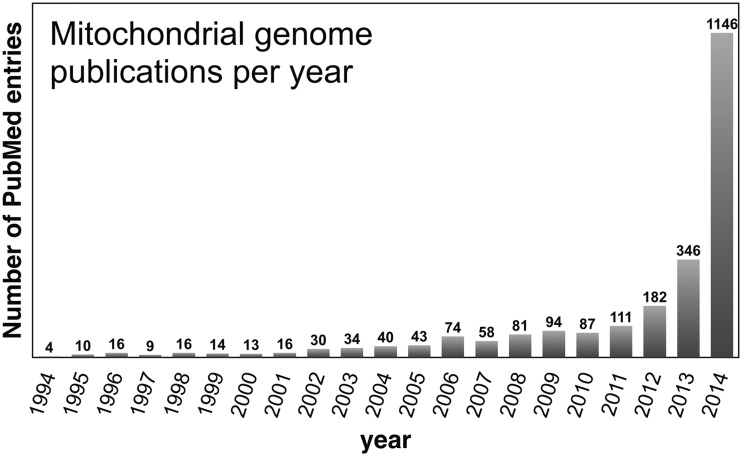
Annual number of peer-reviewed journal articles describing mitochondrial genome sequences. Statistics based on PubMed papers with the words ‘complete mitochondrial genome’, ‘complete mitochondrial DNA’, ‘entire mitochondrial genome’ or ‘mitochondrial genome sequence’ in their title or abstract. These search criteria do not capture all of the published mitochondrial genome sequences per year, but still provide reasonable insights into annual mtDNA publication rate, particularly its large increase over the past 3 years.

There is also an extremely high rate of publication for mitochondrial genomes (Figure 2). In 2014, more than 1100 peer-reviewed articles had the words ‘complete mitochondrial genome’, or a similar derivative, in the title or abstract, exceeding the number from the previous 10 years combined (Figure 2). Many of these articles addressed important and interesting questions, and were covered by major media outlets. For example, a recent issue of the *Guardian Weekly* newspaper (Vol. 192, No. 8) highlighted how scientists are using mtDNA to trace the origin and ancestry of domesticated dogs in the Americas [11]. And there have also been recent innovations in understanding the genetics of mitochondrial diseases [12, 13]. In other instances, however, the articles simply described the mtDNA sequence and its gene content, and did not address a specific hypothesis. Search PubMed for mitochondrial genome papers and you will find a slew of abstracts that begin like this: ‘The complete mitochondrial DNA sequence of… is ∼16 kb long and has 12 protein-, 22 tRNA-, and 2 rRNA-coding genes’. Having sequenced and published many mitochondrial genome papers myself, I am guilty of writing similarly prosaic and formulaic abstracts.

Few would question the utility of mtDNA as a genetic marker, but few would also question that the sequencing of mtDNAs has become a quick and easy route to peer-reviewed publications [14], and at times the pursuit of these publications are encumbering journal editors, referees and the research infrastructure as a whole. Have mitochondrial genomes become a target of the contemporary ‘publish or perish’ academic landscape? Are we still gaining new and significant insights from mitochondrial genome data? Should mtDNAs skip the publication stage and go directly to GenBank? What does the future hold for what are among the most publicized genomes in history?

Here, I address these questions, exploring the past and present impacts of mitochondrial genomes on various fields, and the benefits and drawbacks of continuing to publish mtDNAs at a high rate. I emphasize that for many microbial eukaryotes, mtDNAs are poorly sampled and merit further investigation. I argue that in addition to mtDNA sequencing, more energy could be spent characterizing other aspects of mitochondrial genomes, particularly chromosome structures, transcriptional and translational architectures, population genetics and modes of repair and replication. Although the field is crowded, new technologies and large amounts of publically available eukaryotic NGS data make it an exciting time to be investigating mitochondrial genetics. One, however, needs to be creative and mindful in how they approach, analyse and market these data.

### Early mtDNA discoveries

Our understanding of mitochondria and their genomes is intimately tied to the study of endosymbiosis [15, 16]. Today, it is almost universally accepted that mitochondria arose from the engulfment, retention and integration of a free-living bacterium into a host cell more than 1.5 billion years ago. This theory, however, was once met with widespread scepticism [17], and it was not until the work of Lynn Margulis and others in the 1970s and 1980s, which built on decades of pioneering mitochondrial investigations [18], that the endosymbiotic origin of mitochondria (and chloroplasts) became orthodoxy [19, 20]. Some of the initial compelling observations supporting the endosymbiotic hypothesis included the discovery, in the 1960s, that mitochondria contain DNA and a distinct RNA translation system [15]. Eventually, breakthrough molecular techniques allowed scientists to clone, sequence and characterize mtDNA from a range of species [21]. Together, these findings unequivocally affirmed the bacterial origin of the mitochondrion, which likely has its roots within the phylum α-Proteobacteria [22–24].

The history of mitochondrial research is one of groundbreaking discoveries and monumental achievements, including many in the realm of genomics. In fact, the first non-viral genomes to be completely sequenced were the mouse and human mtDNAs in 1981 (the latter of which helped build momentum towards a human nuclear genome project) [25, 26]. Soon after, complete mtDNAs of bovine [27] and other animals were deciphered [28], which, along with the various partial mtDNA sequences that were available [21], fueled the burgeoning field of comparative genomics. By the early to mid 1990s, dozens of animal mtDNAs had been decoded [29], as well as ones from land plants [30], algae [31], fungi [32] and other protists [33].

Alongside the first wave of mtDNA sequence information were data on other aspects of mitochondrial chromosomes, including their conformations [34, 35], replication strategies and inheritance patterns [36, 37], transcriptional and translational architectures [38], mutational and population genetic landscapes [39, 40] and proclivity for intracellular and lateral gene transfer [41, 42]. In many instances, these data were as challenging to generate as the mtDNA sequences themselves, and provided equal if not greater insights into the evolution and function of mitochondria and eukaryotes. Unfortunately, as discussed further below, investigations into features of mitochondrial genomes apart from the primary nucleotide sequence have not kept pace with mtDNA sequencing.

### Mitochondrial genomics made easy

As Sanger sequencing techniques improved [43], it became easier and more affordable, especially for smaller laboratory groups, to sequence entire mtDNAs. Advancements in sequencing were accompanied by new methods for fast and efficient isolation, amplification, assembly and annotation of mtDNAs [44–48]. For example, enhanced long-range polymerase chain reaction (PCR) techniques allowed for the amplification of entire mitochondrial chromosomes [49], the results of which could then be sequenced stepwise using a ‘primer-walking’ approach, either in-house or through a commercial sequencing centre. Target-specific assembly algorithms (as opposed to all-against-all programs) provided accurate mtDNA assemblies from mixed populations of reads, such as those derived from whole genome eukaryotic shotgun sequencing experiments [50]. Free, user-friendly online software suites, such as the Dual Organellar Genome Annotator [47], supplied ‘one-click’ automated mitochondrial gene prediction services, saving researchers the hassle of sifting through the non-standard genetic codes and eccentric modes of gene expression common to many mitochondria.

Given their proclivity for reduced, circular-mapping structures and conserved coding contents [29], animal mtDNAs were among the easiest organelle genomes to sequence and annotate using these kinds of methods. Accordingly, the bulk of mtDNA data and publications generated during late 90s and start of the new millennium came from metazoans, which is a trend that continues to this day (Figures 1 and 2). Around the same time, scientists were discovering organisms with surprisingly complex mtDNAs [10, 15], which were not amenable to the standard mitochondrial sequencing, assembly and annotation techniques used on most animals. Mitochondrial genomes with huge sizes, fragmented structures, complicated repeats and/or peculiar forms of posttranscriptional processing provided serious challenges to researchers, and in some cases proved to be too complex to accurately characterize [10]. Innovative approaches were sometimes needed to sequence these types of genomes, such as those used to define the mitochondrial telomeres of the green alga *Chlamydomonas reinhardtii* [31] or to assemble the mitochondrial genomic jigsaw puzzle of the euglenozoan *Diplonema papillatum *[6].

As more and more mtDNAs were sequenced, online databanks devoted to the storage, dissemination and description of these sequences emerged. Some of these databanks, including MitoDat [51] and GOBASE [52], have disappeared or are no longer updated, whereas others, such as the Organelle Genome Resources section of GenBank [53] and MITOMAP [54], are still actively maintained and vital assets to the organelle genomic research community. In addition to primary sequence data, these databanks often contain statistics on other aspects of organelle genomes, such as post-transcriptional editing, single nucleotide polymorphisms, gene order, non-canonical genetic codes, and intron insertion sites. Overall, the information within organelle databanks has fueled the field of comparative genomics and made it much easier for researchers to interpret mitochondrial genetic data.

The biggest game-changer in mitochondrial genomics, however, was arguably the introduction of massively parallel sequencing platforms, which are cheaper, faster and can generate orders of magnitude more data than Sanger-based methods [55]. A variety of different NGS techniques, from 454 to Illumina to Ion Torrent, have consistently been used to generate high-quality mitochondrial genome assemblies from whole genomic eukaryotic DNA [2, 56, 57]. High-throughput sequencing has its drawbacks [58], but some of them, such as short read lengths, homopolymer errors and the potential for low read coverage, are not necessarily a problem for mitochondrial genome assemblies [57]. In fact, an oft-cited weakness of eukaryotic NGS experiments is the overabundance of organelle-derived reads relative to nuclear ones, which admittedly is a nuisance for scientists studying nuclear DNA but is helpful for anyone carrying out mitochondrial genomics [2–4].

Besides simplifying and streamlining mitochondrial genomics, NGS and the culture surrounding it has provided a vast reserve of unexplored organelle genetic data. Most journals currently require authors (before publication) to deposit any raw sequencing data used in the study into a publicly accessible repository, such as GenBank’s Sequence Read Archive (SRA) [59]. Given the ubiquity of NGS methods in life science research, these online repositories are accumulating prodigious amounts of sequencing reads from diverse eukaryotic species. As of 1 April 2015, the SRA contained 3.4 quadrillion bases of high-throughput sequencing data. In most cases, the studies and publications employing eukaryotic NGS sequencing ignore the organelle genomes [2]. Thus, the SRA harbours billions of organelle-derived reads from hundreds of different eukaryotes just waiting to be assembled and analysed. During my PhD and postdoc, I took advantage of this fact and mined the SRA for mitochondrial and chloroplast reads. Using data only from the SRA, I assembled and published the complete organelle genomes of more than 10 distinct species, from land plants [60] to algae [61] to jellyfish [62]. In my experience, before publishing analyses based solely on information in the SRA, it is best to contact the primary authors of the data to let them know of your plans and to enquire about potential collaborations.

### The utility of mitochondrial genomes

The mitochondrial genomic data generated over past three decades have impacted a wide swath of scientific fields. Our understanding of eukaryotic life, its origins, its diversity and its complexity have all been shaped by studies of mtDNA [15]. Mitochondrial genes are among the most widely used genetic markers, both for population-level studies [63] and for broad-scale comparative analyses, like those attempting to resolve the eukaryotic tree of life [64]. Some have compellingly argued that the mitochondrial gene *cox1 *should be one of the universal genetic barcodes for eukaryotic biodiversity analyses [65]. Archeologists and forensic scientists have long depended on mtDNA for their work [66, 67], partly because of its uniparental mode of inheritance, high copy number [37] and lower rate of decay relative to nuclear DNA [68]. Medical researchers have used mtDNA as a way to study, diagnose and potentially treat mitochondrial diseases, which are some of the most common types of genetic disorders [12]. Mitochondrial genetics is even becoming an integral part of human reproductive technologies. For instance, cytoplasmic transfer is a modified (and controversial) form of *in vitro* fertilization, which combines the healthy mitochondria of a donor woman with the nuclear DNA of two parents and gives rise to so-called ‘three parent babies’ [13].

More than anything, perhaps, mitochondria have provided an endless reservoir of unconventional genomes. From the enormous, multi-chromosomal mtDNAs of various land plants [69] to the miniature, fragmented mtDNAs of certain alveolates [7] to the baffling chainmail-like mtDNAs of kinetoplastids [70], mitochondrial genomes are anything but ordinary [10]. They have redefined well-established rules in genetics [71], given rise to leading hypotheses on evolution [72], and initiated intense debates about the roles of adaptive versus non-adaptive processes in shaping organismal and genomic complexity [72, 73]. It is hard to imagine the field of molecular evolution as it stands today without the contributions from mitochondrial studies. However, there is also no denying that contemporary mitochondrial research has become crowded, competitive, repetitive and suffers from a general lack of hypothesis testing.

### Too much of a good thing

With state-of-the-art methods for generating complete mtDNA sequences, there came a deluge of publications describing these sequences. The scientific literature is now saturated with mitochondrial genome papers, and has been for sometime (Figure 2). Many of these papers represent the best of what genomics has to offer: they address fundamental questions in biology and are published in top-tier journals [69, 71]. Others, sadly, are unoriginal, add little in terms of new knowledge and reflect more the career-driven obsession with accumulating peer-reviewed papers rather than the progression of science. Of course the same criticisms can be made of all types of genome papers, and there is no denying that the field of genomics as a whole is suffering from a ‘sequence-first-ask-questions-later’ mentality [14]. But because mtDNAs are among the most sequenced chromosomes from across the tree of life, this mindset is particularly pervasive in mitochondrial research. The journal *Mitochondrial DNA *(published by Informa Healthcare) is devoted entirely to the description of mitochondrial genomes. And many popular open-access journals, including *PLoS ONE *and *BMC Genomics*, have become dumping grounds for mtDNA papers of varying quality. All of this is tying up editors, reviewers and the authors themselves, and potentially distracting them from more valuable tasks. However, the publishers of these journals are not complaining, charging fees as high as $2000 USD per paper.

A lot of mitochondrial genome papers are purely descriptive and do not address a specific hypothesis or problem. The value of these kinds of papers is that they let researchers learn about the structure and content of mtDNAs as they become available. In the past, this was important because the GenBank entries containing these sequences were often hard to read and poorly annotated [74]. But now, GenBank entries are usually well annotated—although there is still room from improvement on this front—and there exist powerful, easy-to-use bioinformatics programs for accessing, visualizing and interpreting the entries. Some bioinformatics programs, like Geneious (Biomatters Ltd., Auckland, New Zealand), contain beautiful genome browsers, which allow users to view chromosome maps and extract their annotations and molecular statistics [75]. In my experience, interpreting mitochondrial genome data is often easier using modern bioinformatics software than by reading the primary publication describing the sequence. I would argue, therefore, that in many cases the ‘genome paper’ is no longer needed. The most important thing is annotating the mtDNA correctly and depositing it into GenBank.

Paper or no paper*, *the majority of mtDNAs currently being sequenced come from animals. More than 90% of all mtDNAs in GenBank are metazoan (Figure 1), with the remaining sequences coming primarily from fungi and land plants. Only about 3% of the available mitochondrial genomes are from protists. The proclivity and bias towards sequencing metazoan mtDNAs has undoubtedly helped uncover some interesting genomes, such as the mtDNAs of lice [8]. But this distribution of data does not reflect eukaryotic biodiversity, which is largely microbial and for which animals, fungi and land plants represent only a modest proportion [76]. What is more, common features of metazoan mitochondrial chromosomes, such as a circular-mapping conformation [29] and a high mutation rate [39, 77], are regularly presumed to be representative of *all* organelle DNAs [78, 79]. These presumptions are partly because there is a lack of reference mitochondrial genome sequences from diverse microbial eukaryotes, and non-metazoan eukaryotes as a whole. The paucity of microbial mitochondrial data reflects not only the ‘prioritization’ of animals over other organisms, but also the fact that many microbial eukaryotes are difficult to culture and have complex organelle DNAs, making their mitochondrial genomes hard to sequence. But this may soon be changing.

### Big discoveries from small places: microbial mitochondrial genomics

Recently, there have been major collaborative initiatives to study protist genomics and microbial diversity [80]. The Marine Microbial Eukaryotic Transcriptome Sequencing Project assembled, annotated and made publicly available the transcriptomes from hundreds of diverse marine protists [81]. The raw Illumina sequencing data from these transcriptomes are in GenBank’s SRA and represent an exceptional and untapped resource for studying mitochondrial transcription from some of the most poorly studied, but ecologically important, organisms on Earth. Indeed, large segments of mitochondrial genomes can be transcriptionally active [3], meaning that RNA-Seq results can be mined for both coding and noncoding sequences, facilitating phylogenetic, comparative genomic and genetic barcoding analyses. Moreover, RNA-Seq is an excellent tool for examining the severe and widespread post-transcriptional processing found in various mitochondria [10, 70].

Metagenomics is also aiding microbial mitochondrial research. Massive environmental nucleic acid sequencing projects can capture the genetic information from the viral, prokaryotic and protist microbial communities from which the samples are taken. Organelle genome data are rampant within environmental sequencing projects and new computer algorithms, such as MITObim, are facilitating the assembly of mtDNAs from mixed sequencing samples [82]. Metagenomic data sets now exist for ‘extreme’ and uncharted ecosystems, and the organelle sequences within these projects are helping to uncover previously unknown microbial lineages [83, 84]. It is likely that major new discoveries in mitochondrial research will arise from these and other poorly studied protists, such as *Collodictyon*—an early diverging eukaryotic species [85]—as well as groups that may lack mitochondrial genomes altogether [86].

Protist mtDNAs are renowned for having among the most bizarre features of any genomes [10]. Take, for instance, the euglenozoan *D. papillatum*, whose mtDNA comprises >75 miniature chromosomes, each containing a single gene fragment, which is joined together with its partnered fragments from neighbouring chromosomes through *trans*-splicing [6, 87]. Similarly, the dinoflagellate *Oxyrrhis marina *mtDNA has one of the most reduced gene complements yet described, and the genes that are present are mostly fragmented, fused and/or arranged in tandem copies, and can lack canonical start or stop codons [7]. Moreover, the *O. marina *genome as a whole is found in different arrangements [7]. *Diplonema *and *Oxyrrhis* illustrate the challenges that can be faced when defining protist organelle genomes, but they also underscore the paradigm-shifting discoveries that can come from studying them [10].

Characterizing complex organelle genomes, like those of *Diplonema *and *Oxyrrhis*, often requires more than just DNA sequencing. Gel-electrophoresis, Southern and Northern blotting, quantitative PCR and a slew of other technically demanding and time-consuming molecular techniques are usually needed to accurately describe the architectures of organelle genomes. However, it is exactly these types of analyses that are often lacking from contemporary mtDNA papers. High-throughput sequencing and bioinformatics have removed barriers for obtaining the primary mtDNA sequence. But the primary sequence is often useless if the information on how that sequence is structured, organized and expressed is missing. A scan of the scientific literature shows that many ‘classic’ papers in mitochondrial genetics include extensive gel-electrophoresis, restriction-digest and/or blotting experiments, alongside DNA sequence data [31, 88, 89]. Unfortunately, there appears to be a shift away from these types of well-rounded studies.

The 20 most recently published mitochondrial genome papers in the journal *PloS ONE *(from 16 April 2015) contain solely sequencing-based analyses. Not a single one used molecular techniques other than sequencing to investigate mitochondrial genome architecture, even though it is well documented that the use of genome assembly data alone is a poor predictor of organelle genome structure [78, 79, 88]. With the ease and efficiency of NGS, it is easy to see the appeal of a sequencing-based approach to mtDNA characterization. I am also guilty of taking such an approach. At times I have worked for months with colleagues on obtaining detailed gel-electrophoresis and blotting data on mitochondrial chromosomes, but too often I have just thrown NGS at the problem, and not followed up with complementary analyses. That said, there is a lot of active, cutting-edge mitochondrial genetic research, including the recent structural determination of the mitochondrial ribosome from human and yeast [90, 91].

Moving forward, many eukaryotic species, particularly microbial ones, will require more detailed investigations of their mitochondrial genome architectures, aside from DNA sequencing. In my opinion, there is a need for mitochondrial studies that combine traditional molecular biology techniques, such as pulsed-field gel electrophoresis and blotting, with whole mitochondrial genome sequencing. Research on the chromosome structure, modes of repair, replication and expression and the underlying proteome of mitochondrial systems are also excellent avenues for future research—as are studies on the population genetic and mutational landscapes of organelles. Contemporary studies on mitochondrial genetics are also blending with novel work on bacterial endosymbionts and leading to major advancements in both fields [92]. And work on nuclear genetics is being intertwined with that of mitochondria, leading to a greater understanding of cytonuclear interactions and co-evolution [93]. An emphasis on any of these different research avenues will complement nicely the huge quantity of mitochondrial genome data that are already available and growing ever larger. It may be late in the day for mitochondrial genomics, but the sun has not yet set.

Key points

- Next-generation sequencing techniques have made it quick and easy to sequence entire mitochondrial genomes; consequently, they are the most sequenced and published type of eukaryotic chromosome.
- For many groups, however, mtDNAs are so well sampled that newly published genomes are no longer contributing to the progression of science and are tying up valuable resources.
- In many cases the ‘genome paper’ is arguably no longer needed. The most important thing is depositing the mtDNA into GenBank and annotating it correctly.
- More energy needs to be spent characterizing aspects of mitochondrial genomes apart from the DNA sequence, such as the chromosomal and transcriptional architectures.
- Although the field is crowded, new technologies and large amounts of publically available data make it an exciting time to be investigating mitochondrial genetics. One, however, needs to be creative and mindful in how they approach and market these data.

## Funding

This work was supported by a Discovery Grant to DRS from the Natural Sciences and Engineering Research Council (NSERC) of Canada.
